# Spatio-temporal coherence of circadian clocks and temporal control of differentiation in *Anabaena* filaments

**DOI:** 10.1128/msystems.00700-23

**Published:** 2023-12-11

**Authors:** Rinat Arbel-Goren, Bareket Dassa, Anna Zhitnitsky, Ana Valladares, Antonia Herrero, Enrique Flores, Joel Stavans

**Affiliations:** 1 Department of Physics of Complex Systems, Weizmann Institute of Science, Rehovot, Israel; 2 Department of Life Sciences Core Facilities, Weizmann Institute of Science, Rehovot, Israel; 3 Instituto de Bioquímica Vegetal y Fotosíntesis, CSIC and Universidad de Sevilla, Seville, Spain; Danmarks Tekniske Universitet The Novo Nordisk Foundation Center for Biosustainability, Kgs. Lyngby, Lyngby-Taarbæk, Denmark

**Keywords:** cyanobacteria, circadian clocks, multicellularity, development, *Anabaena*, nitrogen deprivation, single cell analysis

## Abstract

Circadian clock arrays in multicellular filaments of the heterocyst-forming cyanobacterium *Anabaena* sp. strain PCC 7120 display remarkable spatio-temporal coherence under nitrogen-replete conditions. To shed light on the interplay between circadian clocks and the formation of developmental patterns, we followed the expression of a clock-controlled gene under nitrogen deprivation, at the level of individual cells. Our experiments showed that differentiation into heterocysts took place preferentially within a limited interval of the circadian clock cycle, that gene expression in different vegetative intervals along a developed filament was discoordinated, and that the circadian clock was active in individual heterocysts. Furthermore, *Anabaena* mutants lacking the *kaiABC* genes encoding the circadian clock core components produced heterocysts but failed in diazotrophy. Therefore, genes related to some aspect of nitrogen fixation, rather than early or mid-heterocyst differentiation genes, are likely affected by the absence of the clock. A bioinformatics analysis supports the notion that RpaA may play a role as master regulator of clock outputs in *Anabaena*, the temporal control of differentiation by the circadian clock and the involvement of the clock in proper diazotrophic growth. Together, these results suggest that under nitrogen-deficient conditions, the clock coherent unit in *Anabaena* is reduced from a full filament under nitrogen-rich conditions to the vegetative cell interval between heterocysts.

**IMPORTANCE:**

Circadian clocks, from unicellular organisms to animals, temporally align biological processes to day and night cycles. We study the dynamics of a circadian clock-controlled gene at the individual cell level in the multicellular filamentous cyanobacterium *Anabaena*, under nitrogen-stress conditions. Under these conditions, some cells along filaments differentiate to carry out atmospheric nitrogen fixation and lose their capability for oxygenic photosynthesis. We found that clock synchronization is limited to organismic units of contiguous photosynthetic cells, contrary to nitrogen-replete conditions in which clocks are synchronized over a whole filament. We provided evidence that the circadian clock regulates the process of differentiation, allowing it to occur preferentially within a limited time window during the circadian clock period. Lastly, we present evidence that the signal from the core clock to clock-regulated genes is conveyed in *Anabaena* as in unicellular cyanobacteria.

## INTRODUCTION

Circadian clocks arose during evolution to enable organisms, from cyanobacteria to plants and mammals, to tune their metabolism and bioprocesses to daily light/darkness cycles on Earth and thereby optimize their fitness (1, 2). Much of what is known about the mechanisms behind circadian clocks in the case of cyanobacteria has been learned from investigations of unicellular organisms, primarily *Synechococcus elongatus* strain PCC 7942 (henceforth *S. elongatus*). These investigations have firmly established that the core clock is comprised of three proteins, KaiA, KaiB, and KaiC, the first two of which modulate the four phosphorylation states of KaiC, which cycle with time. The information encoded in the phosphorylation state of KaiC is then relayed to clock-controlled genes by the master transcription factor RpaA (3, 4) via the input/output sensor histidine kinases CikA and SasA, the phosphatase and kinase that modulate RpaA activity (5). KaiA and KaiB regulate the phosphorylation state of KaiC in a negative feedback loop configuration that drives the oscillatory gene expression. In addition to the elucidation of many mechanistic details (5), other investigations have provided evidence indicating that the circadian clock in *S. elongatus* gates the cell cycle (6, 7) and regulates the competence state, natural transformation being maximal when the onset of darkness coincides with the dusk circadian peak (8).

An important cyanobacterial order, *Nostocales*, consists of multicellular organisms such as *Anabaena* sp. strain PCC 7120 (henceforth *Anabaena*, also known as *Nostoc*), in which cells are organized in a filamentary structure, with local, nearest-neighbor cell-cell coupling via septal junctions (9). *Anabaena* bears homologs not only of the core *kai* genes of *S. elongatus* (10) but also of the network of genes in which they are embedded and those coding for RpaA, as well as CikA and SasA. Whereas not much is known about the detailed molecular mechanisms behind the circadian clock in *Anabaena*, structural studies suggest that the interactions between the respective proteins are similar (11).

First insights into the dynamical behavior of clocks in *Anabaena* were obtained from bulk and DNA microarray studies that established that its circadian clock is autonomous and that it can run freely under constant light conditions (12). Following entrainment by two 12-h light-dark cycles, a group of genes exhibiting oscillatory behavior were identified, and the homologs of *kaiA*, *kaiB*, and *kaiC* genes showed low-amplitude or arrhythmic expression, in contrast to those of *S. elongatus* (12). More recently, the collective behavior of circadian clocks in *Anabaena* filaments has been studied at the individual cell level (13). Circadian clocks along filaments were interrogated under nitrogen-replete conditions in which all cells in the filaments were vegetative, carrying out both oxygenic photosynthesis and assimilation of a source of combined nitrogen. Under these conditions, filaments grow by binary fission of each and every cell along their length. This study found significant synchronization and spatial coherence of clock phases on the scale of filaments, evidence supporting the notion of clock coupling via cell-cell communication, and gating of the cell division by the circadian clock. Furthermore, the study confirmed the low-amplitude circadian oscillatory transcription of *kai* genes comprising the post-transcriptional core oscillatory circuit suggested by results of a bulk study (12) and found evidence of large-amplitude oscillations of *rpaA* transcription (13).

Under nitrogen-deficient conditions, *Anabaena* fixes atmospheric nitrogen, an activity that is incompatible with the oxygen produced by photosynthesis (14). The incompatibility of photosynthesis and nitrogen fixation is solved by division of labor: filaments undergo a process of development into a one-dimensional pattern consisting of single, specialized micro-oxic cells, the heterocysts, in which atmospheric nitrogen fixation takes place, separated by about 10–15 vegetative cells that fix CO_2_ photosynthetically (9, 15, 16). The genetic cascade leading to heterocyst formation is controlled by the master regulator of differentiation HetR and involves at least two inhibitory signals related to the PatS polypeptide and the HetN protein that can be transferred by cell-cell communication. Heterocyst differentiation and the ensuing emergence of developmental patterns in *Anabaena* entail profound metabolic and morphological changes (9, 17), including some that affect cell-cell communication (18). Results from a DNA microarray analysis of heterocyst-enriched samples have provided evidence of circadian clock activity of *kai* genes in heterocysts (12). However, the possibility that the rhythmic transcription was indirectly induced in heterocysts by time-dependent intercellular signals from oscillators in neighboring vegetative cells could not be excluded. Moreover, the results revealed that under nitrogen-deficient conditions, 39 of the 78 previously identified clock-controlled genes preserved rhythmic expression, a subset being heterocyst specific (12). Of note, the number of genes reported to oscillate with a circadian period in *S. elongatus* is 856, significantly larger than 78 (4, 19).

Here, we set out to study the interplay between the circadian clock and the genetic network controlling heterocyst differentiation under nitrogen-deficient conditions in *Anabaena*. We tracked circadian rhythms in individual vegetative cells and heterocysts in combined nitrogen-deprived *Anabaena* filaments in real time, by following the expression from the promoter of *pecB*, a clock-controlled gene that exhibits high-amplitude oscillations (12). This gene is part of the *pecBACEF* operon and codes for the beta subunit of phycoerythrocyanin, a structural component of the phycobilisome rod that plays a major role in light harvesting for photosynthesis (20). Our study provides evidence that nitrogen deprivation has a profound influence on the synchronization and spatial coherence of clocks along a filament and that in addition to possible gating the cell cycle, the circadian clock also influences the timing of cellular differentiation.

## MATERIALS AND METHODS

### Strains

The construction of strains bearing a chromosomally encoded P*_pecB_-gfp* was obtained by conjugation with wild-type or mutant *Anabaena* sp. (also known as *Nostoc* sp. PCC 7120). Strains carrying P*_pecB_-gfp* in wild type (WT) background and an *Anabaena* deletion mutant of the *kaiABC* genes, bearing a *pecB* promoter fusion to gfp (P*_pecB_-gfp*, Δ*kaiABC*), were described previously (13). The genetic structure in the Δ*kaiABC* mutants is summarized in Fig. S3.

### Culture conditions

Strains were grown photoautotrophically in BG11 medium (containing NaNO_3_) supplemented with 20 mM HEPES (pH 7.5) with shaking at 180 rpm, at 30°C, as described previously (21, 22). Growth took place under constant illumination (10 µmol of photons m^−2^ s^−1^; spectrum centered at 450 nm) from a cool-white LED array. For the strain carrying P*_pecB_-gfp* in a wild-type background, streptomycin sulfate (Sm) and spectinomycin dihydrochloride pentahydrate (Sp) were added to the media at final concentrations of 2 µg/mL for liquid and 5 µg/mL for solid media (1% Difco agar). For the Δ*kaiABC* strain, neomycin sulfate (Nm) was added at 10 and 25 µg/mL for liquid and solid media, respectively. The densities of cultures were adjusted so as to have a chlorophyll *a* content of 2–4 μg/mL 24 h prior to the experiment, following published procedures (16). For time-lapse measurements, filaments in cultures were harvested and concentrated 50-fold.

### Samples for time-lapse microscopy

Strains were grown as described previously (16). The densities of the cultures, grown under an external LED array (15 µmol m^−2^ s^−1^) for about 5 days, were adjusted to have a chlorophyll *a* content of 2–4 μg/mL 24 h prior to the experiment following published procedures (16). For time-lapse, single-cell measurements of *Anabaena*, 5 µL of culture concentrated 100-fold was pipetted onto an agarose low-melting gel pad (1.5%) in BG11 medium containing NaNO_3_ and 10 mM NaHCO_3_, which was placed on a microscope slide. The pad with the cells was then covered with a #0 mm coverslip and then placed on the microscope at 30°C. The cells grew under continuous light from both an external LED array (15 µmol m^−2^s^−1^) and tungsten halogen light (10 µmol m^−2^s^−1^), 3,000 K color), except when images were taken. Under these illumination conditions, the doubling time of cells is similar to that in bulk cultures (16). The change in illumination conditions when transferring cells from bulk cultures to the microscope results in high synchronization within filaments. Images of about 10 different fields of view were taken every 30 min on a Nikon Eclipse Ti-E microscope controlled by the NIS-Elements software using a 60 NA 1.40 oil immersion phase-contrast objective lens (Nikon plan-apochromat 60 1.40) and an Andor iXon X3 EMCCD camera. Focus was maintained throughout the experiment using a Perfect Focus System (Nikon). All the filters used are from Chroma. The filters used were ET480/40× for excitation, T510 as dichroic mirror, ET535/50M for emission (GFP set for green fluorescent protein), ET500/20× for excitation, T515lp as dichroic mirror, and ET535/30 m for emission (EYFP set), and ET430/24× for excitation, 505dcxt as dichroic mirror, and HQ600lp for emission (chlorophyll set). Samples were excited with a pE-2 fluorescence LED illumination system (CoolLED).

### Image segmentation

All image processing and data analysis were carried out using Matlab (MathWorks). Filament and individual cell recognition were performed on phase contrast images using an algorithm developed in our laboratory. The program’s segmentation was checked in all experiments and corrected manually for errors in recognition. The total fluorescence from GFP and chlorophyll *a* (autofluorescence) channels of each cell, as well as the cell area, were obtained as output for further statistical analysis.

### Analysis of synchronization along filaments

Synchronization was measured by the synchronization index *R* (23):


(1) $$R=\frac{\left\langle \mu^{2}\right\rangle -{\left\langle \mu \right\rangle }^{2}}{\bar{\left\langle f_{i}^{\text{2}}\right\rangle -{\left\langle f_{i}\right\rangle }^{\text{2}}}}$$

where ⟨⋅⟩ denotes a time average, the upper bar $\bar{\cdot }$ indicates an average over all cells, and μ denotes the average of the fluorescence intensity of each cell $f_{i}$ along a filament. Hence, R is defined as the ratio of the variance of μ(t) over a cycle to the variance of individual cell fluorescence intensities $f_{i}$, averaged over all cells along a filament. For measurement of synchronization within a filament, groups of 8–11 cells were chosen, either contiguous or separated near the centers of different vegetative intervals. All the evaluations of R were carried out over a full period of oscillation. The final result comprises the mean of at least three independent repeats, in at least two independent experiments.

### Determination of the phase of the onset of differentiation along a circadian cycle

The onset time of the decay of autofluorescence (AF) of photosynthetic pigments in an incipient heterocyst was determined as the stitching point of a piecewise fit consisting of a constant signal (before the onset) and a parabolic fit to the initial stages of the decay (after the onset). The corresponding phase of the onset time along the cell’s circadian cycle was defined as the fraction between this time, measured relative to the last minimum of a circadian cycle of P*_pecB_-gfp* expression, and the difference of times between this minimum and the consecutive one. Minima of circadian cycles were determined by fitting the fluorescence intensity from P*_pecB_-gfp* expression with either a parabolic fit or by piecewise fits of a parabola and a linear polynomial. The errors in the determination of either the onset of decay of AF or the minima of circadian cycles do not exceed 1 h (two frames) and are subsumed in the bin widths of the phase histogram (Fig. 4, main text).

### Identification of RpaA binding motifs in the *Anabaena* genome

Motif scanning was done using FIMO tool from the MEME Suite (v5.4.1) (24), using a previously reported DNA Position-specific probability matrix of the RpaA binding motif in *S. elongatus* (4), scanning both strands and reporting a minimal match *P*-value of 10^−4^. The results were restricted to sequences within a window of −500 bp to +50 bp relative to the transcription start site (TSS), based on previously reported *Anabaena* TSS annotations (25). Assignment of resulting motifs to genes was based on annotations of valid gene names (25), as well as reported annotations of early and late differentiation genes [cluster 6 and cluster 4 genes (12, 26)]. Protein sequences coded by *Anabaena* genes, which harbor a putative RpaA motif, were compared with genes reported to bind RpaA in a chromatin immunoprecipitation with high-throughput sequencing (ChIP-Seq) experiment in *S. elongatus* (4). Thus, protein sequences of 89 reported PCC 7942 genes were compared using BLASTP to *Anabaena* proteins, reporting hits with E-value ≤0.005 and >36% sequence similarity (Table S2).

## RESULTS

### Discoordinated expression of a clock-controlled gene along filaments under constant light conditions

**Fig 1:**
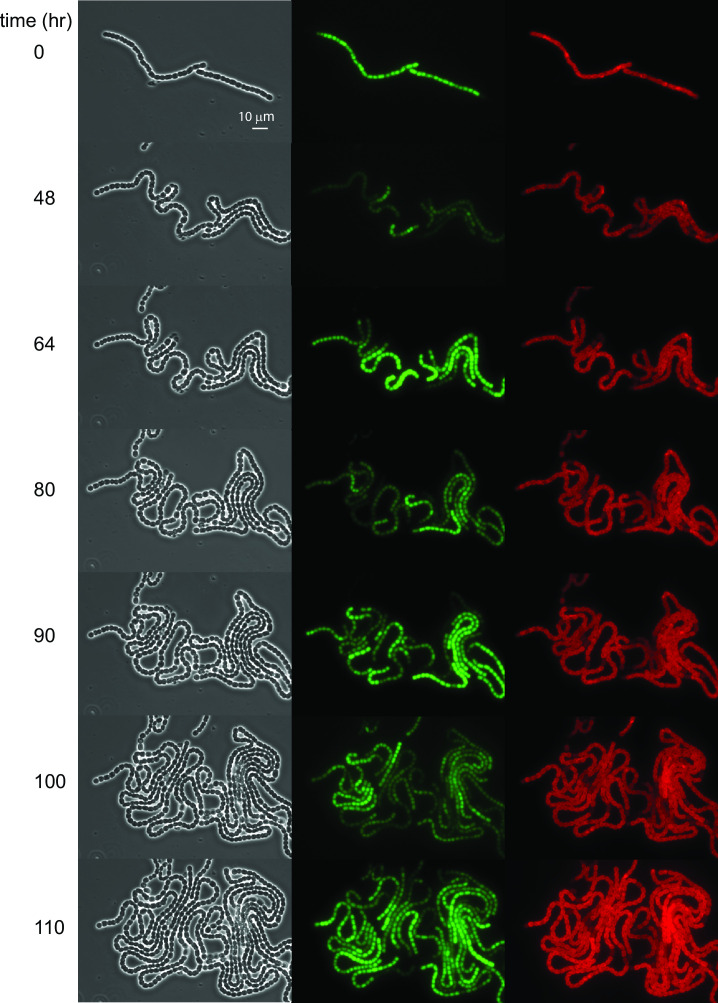
Circadian oscillations in *Anabaena* filaments under nitrogen-poor conditions. (Left) Phase contrast images of a filament of an *Anabaena* strain bearing a P*_pecB_-gfp* promoter fusion, growing under nitrogen-poor conditions. (Middle) GFP fluorescence of the same *Anabaena* filament growing under nitrogen-poor conditions. (Right) Autofluorescence as a function of time of the same *Anabaena* filament. Snapshots correspond to those in the left-hand micrographs, and time 0 corresponds to the time at which filaments were placed under the microscope. The times at which snapshots were taken were chosen near maxima and minima of the circadian oscillations observed in GFP fluorescence intensity. For a time-lapse movie, see Movie 1.

The *pecB* gene, encoding the beta subunit of phycoerythrocyanin (20), is known to display circadian oscillations both under nitrogen-replete and nitrogen-deficient conditions (12). Expression from a chromosomal fusion of *gfp* to the 5′ region of *pecB*, denoted as P*_pecB_-gfp* (13), was followed along wild-type *Anabaena* filaments under constant light, after submitting filaments to nitrogen deprivation in BG11_0_ medium. A series of phase contrast, fluorescence, and autofluorescence of photosynthetic pigment (AF) snapshots taken at maxima and minima of a number of circadian cycles is shown in Fig. 1. In contrast to the images taken right after nitrogen deprivation (*t* = 0), in which expression along a filament was largely uniform except for small amplitude variations, at later times filaments displayed considerable heterogeneity. This heterogeneity may be due to demographic noise or, alternatively, may reflect different metabolic states in different cell stretches of the filament (27). Expression from P*_pecB_-gfp* was visibly higher in some vegetative intervals than in others, alternating in time, and the heterogeneity in expression was spatially locked with the instantaneous pattern of heterocysts to a large extent (Fig. 1; Movie 1).

**Fig 2:**
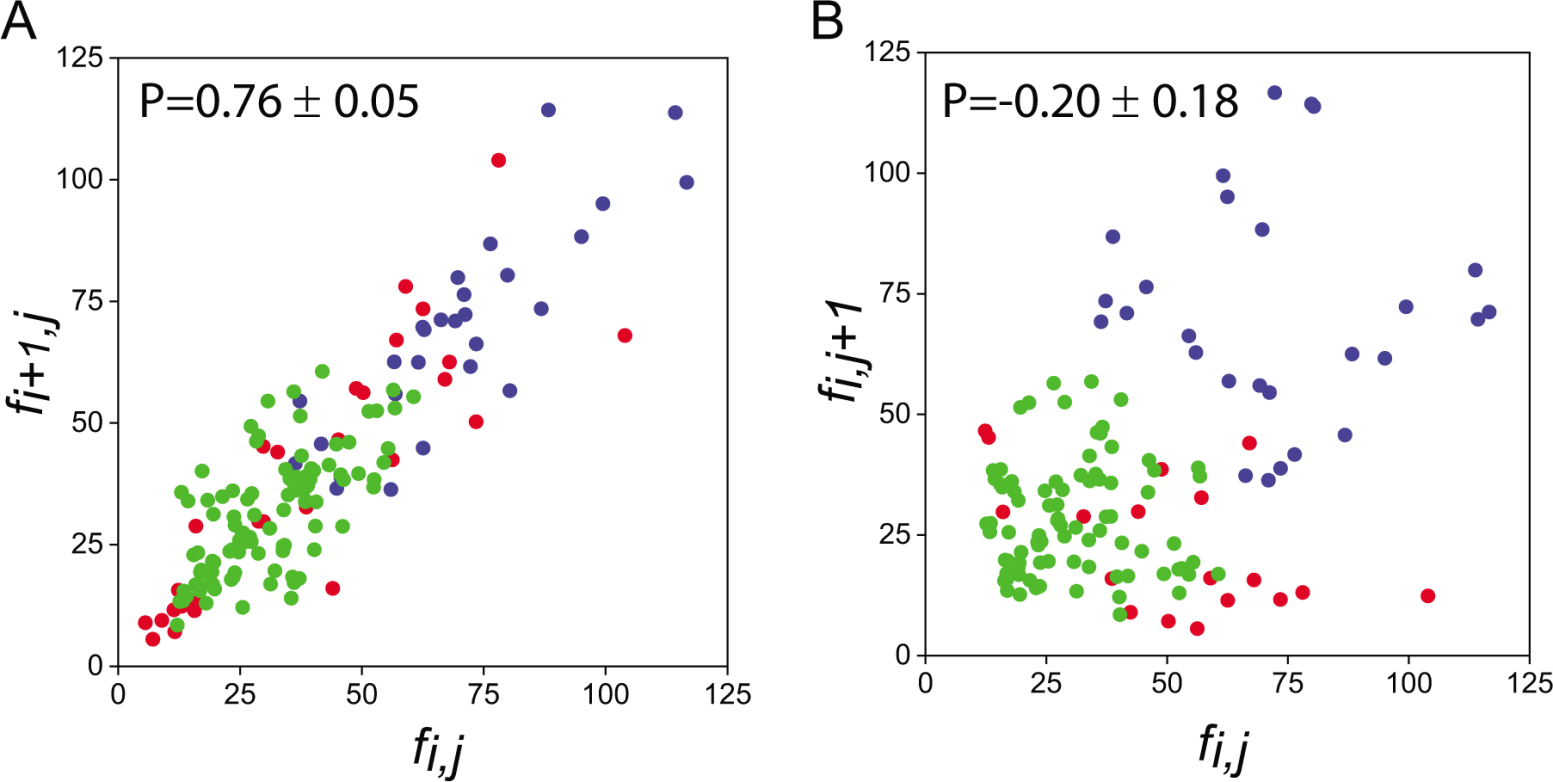
Discoordinated expression of the *pecB* promoter P*_pecB_-gfp* between vegetative cell intervals. (**A**) Scatter plot of the fluorescence intensities of nearest-neighbor cells ${(f}_{i,j},f_{i+1,j})$ within vegetative cell intervals j. (**B**) Scatter plot of the fluorescence intensities of corresponding cells in adjacent vegetative cell intervals ${(f}_{i,j},f_{i,j+1})$. Each color in A and B represents data from three independent experiments. Data were taken from images corresponding to one-fourth or three-fourth of the circadian cycle period, when fluctuations in expression are maximal (13).

The physiological changes involved in the differentiation of a vegetative cell into a heterocyst entail alteration of cell-cell communication (9, 28). To test the notion that altered communication may affect the spatial coherence along a filament, we made scatter plots of the fluorescence intensities $f_{i,j}$ of nearest-neighbor cells along vegetative intervals, where i indexes cells along vegetative cell interval j (Fig. 2A). The scatter plot displays a distinct cigar shape, and the Pearson correlation coefficients for each experiment are high (*P* = 0.75 ± 0.06, mean ± SE, *n* = 3). Of note, under nitrogen-replete conditions, the fluorescence intensities along a whole filament are much more uniform, yielding nearest-neighbor scatter plots with tight clusters (Fig. S1). In contrast, scatter plots of fluorescence intensities of corresponding cells in adjacent vegetative intervals of ($f_{i,j+1}$ versus $f_{i,j}$) exhibit widely spread clusters characterized by small values of the Pearson coefficient whose mean is *P* = −0.20 ± 0.18 (mean ± SE, *n* = 3) (Fig. 2B). Thus, cells in different intervals tend to have a wide distribution of circadian clock phases. An alternative characterization of the loss of spatial coherence in expression when filaments are subject to nitrogen-poor conditions can be obtained by estimating the cell-to-cell variability or noise, defined as the square of the coefficient of variation (standard deviation divided by the mean) of the intensity of all vegetative cells along a filament, of both expression from P*_pecB_-gfp* and AF, respectively (Fig. S2). Also shown is the noise evaluated over individual vegetative intervals and then averaged over a number of these. There are a number of salient features. First, the mean noise over an interval (0.20 ± 0.13) is not significantly larger than the noise evaluated over all vegetative cells along the same filament (0.12 ± 0.07). Second, the latter is larger than for nitrogen-replete conditions (0.054 ± 0.02) (Fig. S2). Lastly, the noise of autofluorescence intensity (0.008 ± 0.005) is significantly smaller than that of P*_pecB_-gfp* expression.

To quantitate the extent of synchronization, the value of a synchronization index *R* was calculated for all cells within a vegetative interval (Table 1) and averaged over many such intervals (see Materials and Methods). The obtained value, *R* = 0.77 ± 0.03, was somewhat smaller than the value obtained for contiguous cells in nitrogen-rich filaments (13), *R* = 0.89 ± 0.04. To probe large-scale synchronization, we calculated the value of *R* for a group of individual cells, each near the center of a different vegetative interval (Table 1), and obtained *R* = 0.39 ± 0.09 (from three independent experiments), well below the value obtained for well-separated cells along filaments under nitrogen-replete conditions (13) (*R* = 0.85 ± 0.01). These findings indicate that, in diazotrophic filaments, oscillations in vegetative intervals are desynchronized from one another, much like cells from entirely separate filaments, but maintain a normal degree of synchrony within intervals.

**TABLE 1:** Synchronization index R of vegetative cells under nitrogen-poor conditions^*a*^

Cell cluster	*R* (mean ± SEM)	Cells over which *R* was calculated
Contiguous vegetative	0.77 ± 0.03	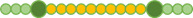
Separated vegetative	0.39 ± 0.09	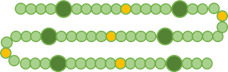

^*a*^ The synchronization index *R* (Materials and Methods) of vegetative cells within filaments under nitrogen-poor conditions is shown. First row: all contiguous vegetative cells within heterocyst-bounded intervals (at least eight cells per interval and two independent intervals). Second row: vegetative cells separated by a heterocyst (at least eight cells, three independent experiments). Yellow cells in the respective cartoons represent the vegetative cells in filaments over which *R* was calculated in each case. Dark green cells represent heterocysts, and light green represent vegetative cells.

### Sequential turnoff of expression in vegetative cells between consecutive heterocysts

**Fig 3:**
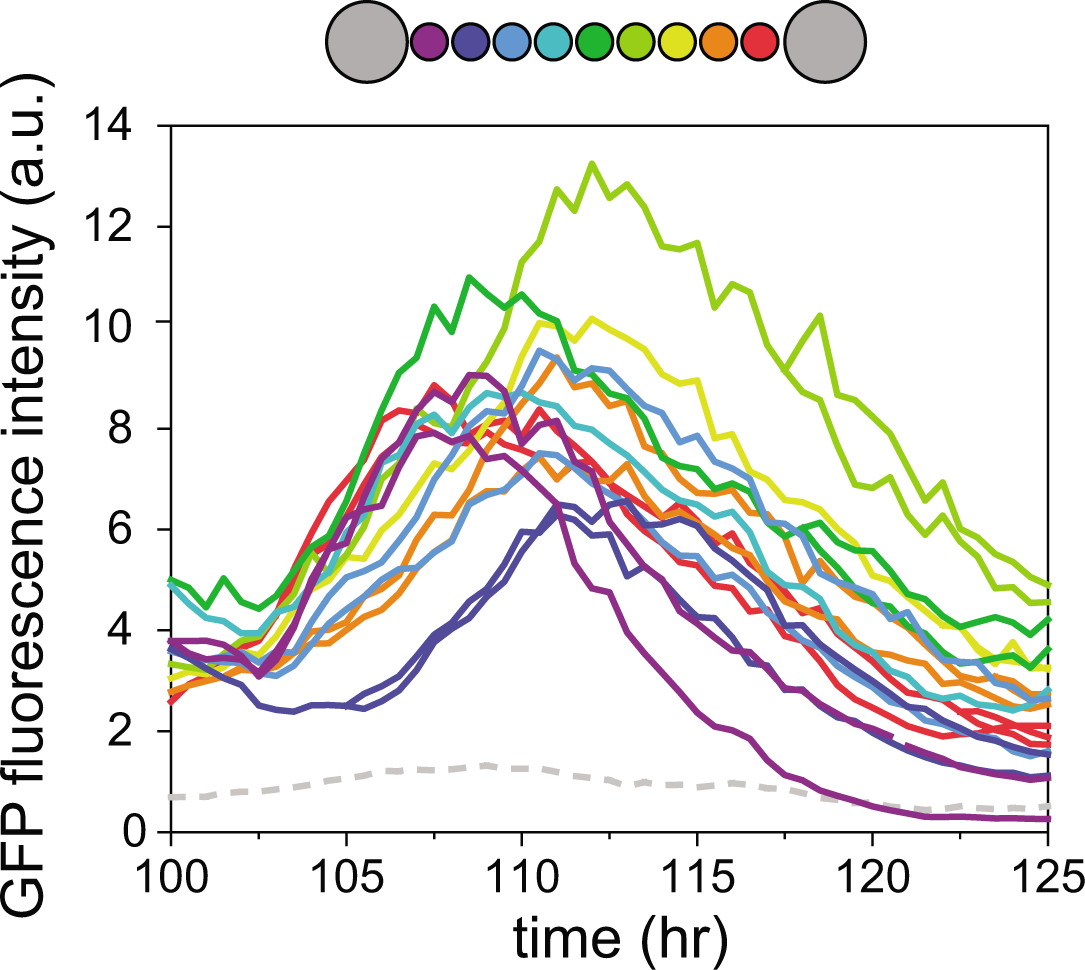
Gradient and sequential activation of fluorescence intensity from P*_pecB_-gfp* of all vegetative cells within a heterocyst-bound interval. Representative fluorescence intensity of individual cells as a function of time over one circadian cycle in arbitrary units. Traces are color coded according to their position along a heterocyst-bound vegetative interval as illustrated in the schematic filament above. Daughter cells are colored as their mother. The gray dashed line at the bottom of the plot corresponds to the trace of one of the bounding heterocysts. This cycle corresponds to the third cycle in Fig. 5B.

A salient feature of oscillations in the fluorescence intensity from P*_pecB_-gfp* was the cell-cell variation in the times at which expression was turned off in vegetative cell intervals, preempting the decrease of the fluorescence intensity and the completion of a cycle. This decrease, mediated presumably both by dilution by cell growth and degradation of the GFP, resulted in a particularly wide spread of fluorescence intensity values between cells. To check whether there is coordination in expression turnoff times along a filament, we represented the fluorescence intensity in individual contiguous cells in a heterocyst-bound interval during one oscillation, color-coded according to their spatial position along the vegetative interval (Fig. 3). Notably, the fluorescence intensity during upregulation was synchronized among cells, but the decay was delayed as a function of the cell’s distance to a heterocyst. Thus, the decay in cells near the middle of a vegetative interval was most delayed. Note also that cells near the middle of the vegetative interval appear to display higher expression. This behavior was observed in different filaments, times, and independent experiments (see also Fig. 1). We did not detect sequential turnoff under nitrogen-replete conditions.

### Heterocyst differentiation is restricted to a narrow window of the circadian cycle

**Fig 4:**
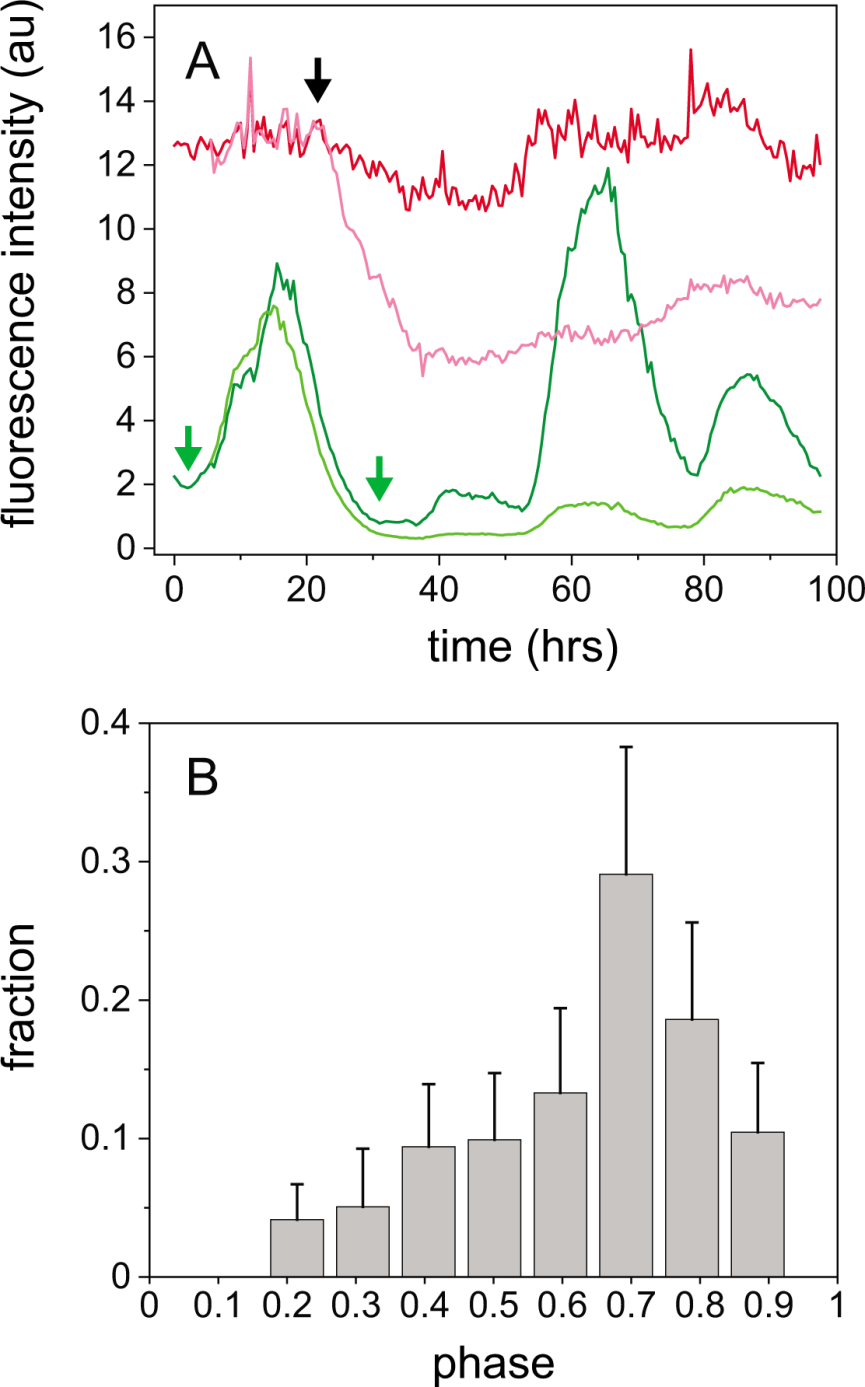
Restriction of heterocyst differentiation by the circadian clock to a narrow temporal window. (**A**) Fluorescence intensities of two neighboring, sister cells bearing a P*_pecB_-gfp* fusion (green) one of which eventually becomes a heterocyst, and their respective autofluorescence intensity traces (red) as a function of time, under conditions of constant illumination. Light green and red lines correspond to the cell that becomes a heterocyst. The onset in the decay of AF in the cell that becomes a heterocyst is indicated with a black arrow, whereas the positions of the circadian cycle minima on either side are indicated with green arrows. (**B**) Normalized histogram of the phase of onset times of autofluorescence reduction in cells that become heterocysts, with 0 and 1 denoting two consecutive minima in circadian cycles in units of 2π. Data from three independent experiments and *n* = 45 differentiation events, observed from the second to the fourth cycles. Error bars were determined from 1,000 bootstrap samples.

To test whether the heterocyst differentiation process and circadian clocks are temporally coordinated in *Anabaena* cells, we determined the onset of the reduction of the autofluorescence of photosynthetic pigments (AF) in a cell that eventually will become a heterocyst as a temporal reference point (29–31) and its phase along the cell’s circadian cycle, taking 0 and 2π to correspond to consecutive minima in the cyclic expression from P*_pecB_-gfp* (Fig. 4A; Materials and Methods). A histogram of the phases of AF intensity reduction events obtained from traces similar to those in Fig. 4A is shown in Fig. 4B. Clearly, the initiation of differentiation takes place within a narrow temporal window of the circadian cycle. We suggest that the circadian clock establishes a temporal control or gates heterocyst differentiation. Lastly, we note that the phase of oscillation in the heterocyst was similar to the phase of its vegetative sister, suggesting that it was inherited from that of the original vegetative cell.

### Circadian oscillations in heterocysts

**Fig 5:**
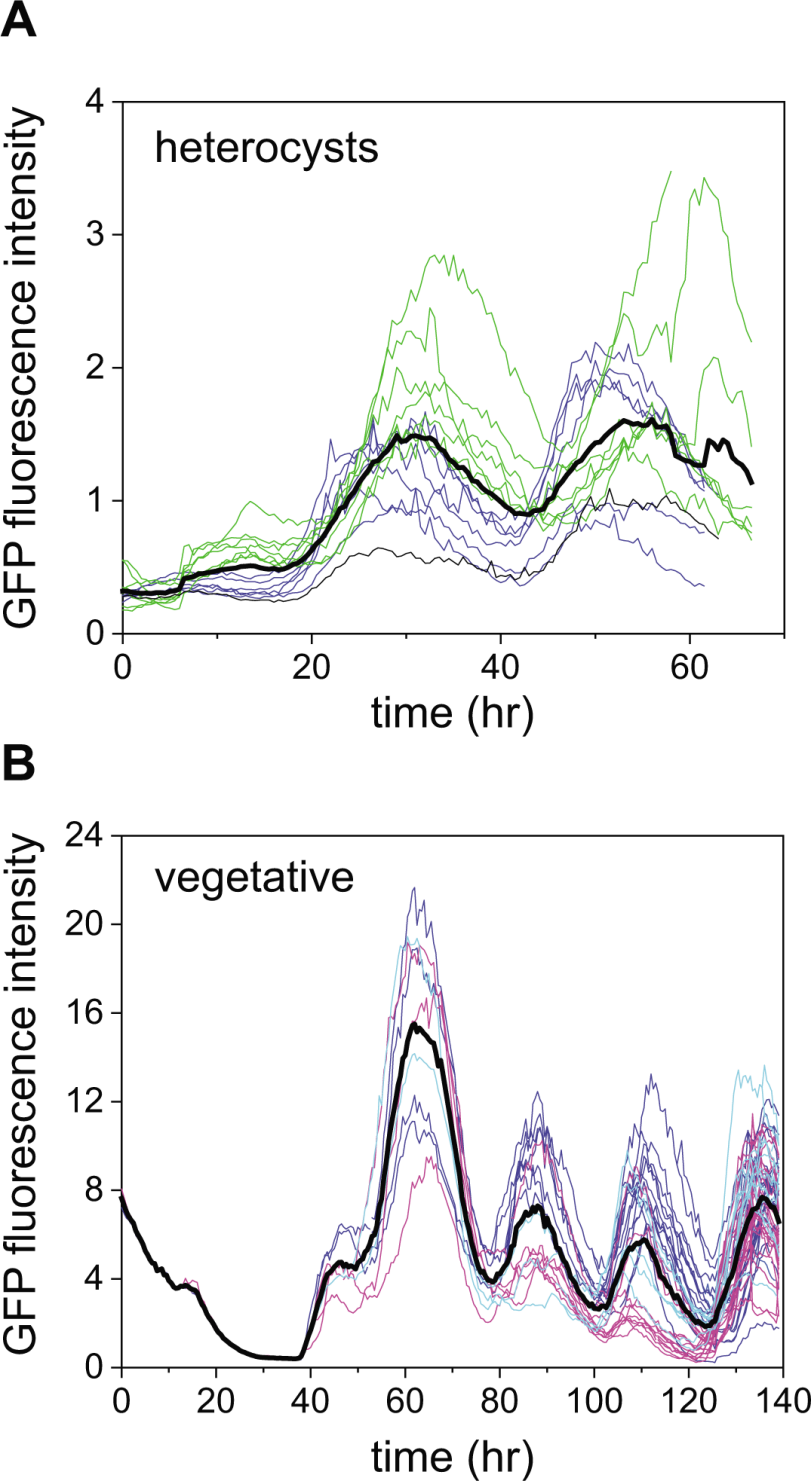
Fluorescence intensity from P*_pecB_-gfp* as a function of time in heterocysts and vegetative cell lineages. (**A**) Fluorescence intensity in heterocysts. Different colors correspond to data from different filaments. Traces were displaced so that the onsets of decay of autofluorescence in the vegetative cells that differentiate into heterocysts coincide. (**B**) Lineages of three cells each in one of three contiguous vegetative cell intervals (color coded according to their respective interval). Thick black lines in both panels correspond to the average of all traces, and the fluorescence intensity is in arbitrary units.

Evidence for circadian clock activity in heterocysts was obtained previously by interrogating heterocyst-enriched bulk samples (12). However, these experiments could not exclude the possibility that oscillations were induced in heterocysts by neighboring vegetative cells. To test whether oscillatory behavior takes place in heterocysts, we followed expression from P*_pecB_-gfp* in individual heterocysts that formed after filaments were subjected to nitrogen deprivation (incubation in BG11_0_ medium). The fluorescence intensity of individual heterocysts in a typical experiment is shown in Fig. 5A. Since heterocysts formed at different times during the experiment, traces have been temporally aligned by using the onset of the decay in the autofluorescence as a temporal reference point (29–31) (e.g., Fig. 4A). A comparison of these traces with those of three vegetative cells and their respective lineages (Fig. 5B) shows that the period of the oscillations in heterocysts (21.3 ± 0.6, mean ± SE, *n* = 30) is undistinguishable from that observed in vegetative cells (20.8 ± 0.4, mean ± SE, *n* = 35) and that their amplitude is about a factor of five smaller. The lower amplitude may result from lower expression from the *pecB* promoter under N-poor conditions (18) that may correspond to repression in heterocysts, which specifically loose pigments compared to vegetative cells (32). It is remarkable that the oscillation of fluorescence from P*_pecB_-gfp* was clearly observed in differentiating or mature heterocysts in spite of the decreased activity of the P*_pecB_* promoter.

### Δ*kai* mutant filaments fail to grow fixing N_2_

**Fig 6:**
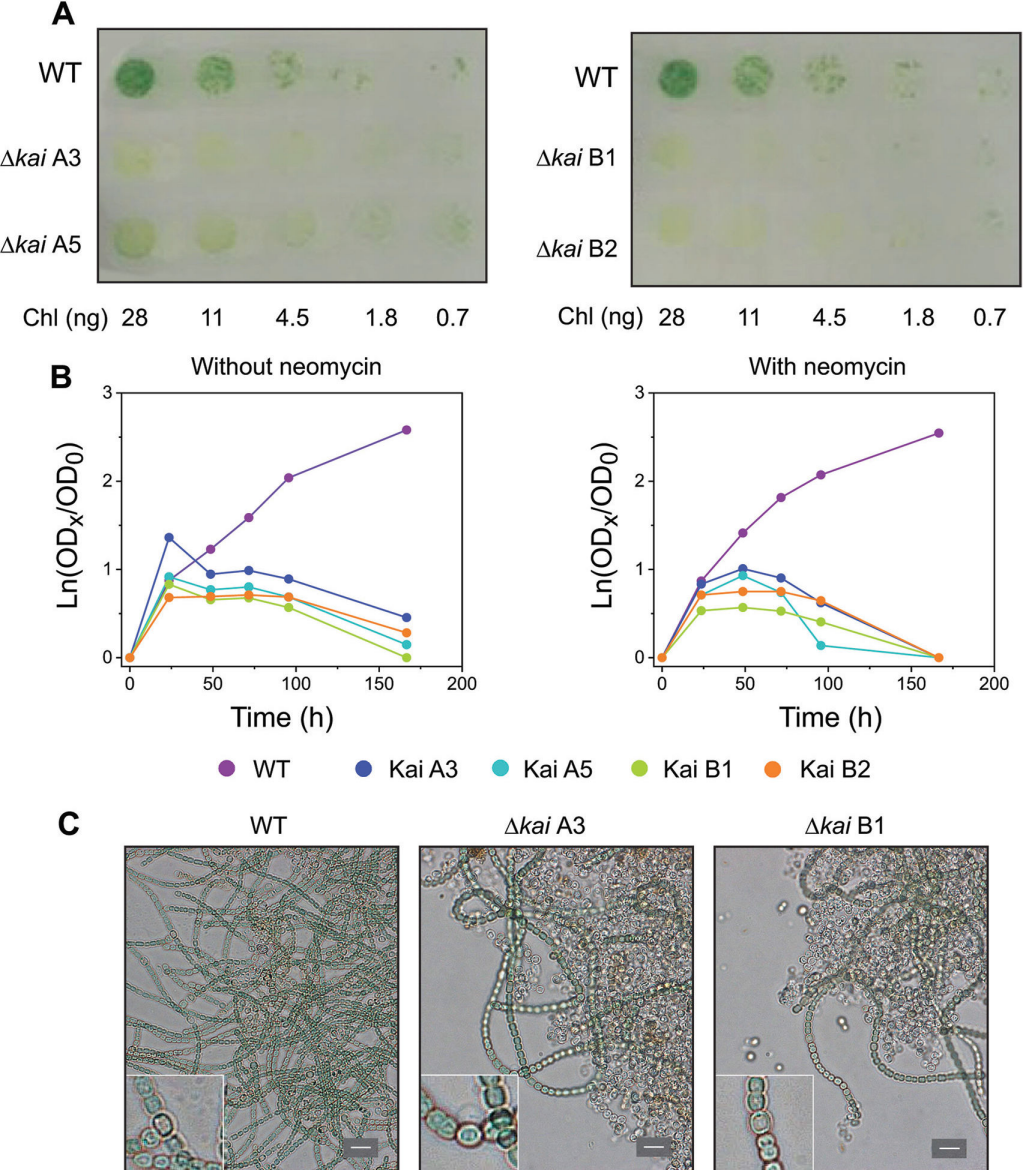
Phenotype of the Δ*kai* mutants of *Anabaena* sp. strain PCC 7210. (**A**) Growth tests on plates with BG11_0_ medium. The filaments were grown in BG11 medium with neomycin at 20 µg mL^−1^, washed with BG11_0_ medium (without neomycin), and incubated in BG11_0_ medium (without neomycin) for 10 days under photoautotrophic culture conditions (constant light). (**B**) Growth tests in liquid BG11_0_ medium without neomycin or with neomycin at 20 µg mL^−1^ for the mutants, as indicated. y-axis, Ln (OD_750_ nm at time X/OD_750 nm_ at time 0); x-axis, time of incubation under photoautotrophic culture conditions (constant light). For panels A and B, the WT and two mutant clones from each orientation of the gene cassette were analyzed. (**C**) Brightfield micrographs of the WT and Δ*kai* mutant (clones A3 and B1) after 10 days of incubation in BG11_0_ medium without antibiotic. Whereas the WT formed long filaments, much cell debris and only a few filaments were observed for the mutants. Size bar, 10 µm. Insets, further magnification (5×) showing the presence of heterocysts in the three strains.

To understand further the role played by the circadian clock in filament behavior under nitrogen deprivation, we studied the phenotype of filaments in which *kaiABC* genes were deleted (henceforth Δ*kaiABC* strains; mutant construction is schematically shown in Fig. S3). The growth of Δ*kaiABC* strains was studied using four independent clones: two in which the C.K1 gene cassette was inserted in direct orientation (clones A) and two in which it was inserted in reverse orientation (clones B) with regard to the orientation of the operon. None of the clones could grow under photoautotrophic conditions in solid BG11_0_ medium, which lacks combined nitrogen (Fig. 6A). In liquid BG11_0_ medium, after an initial increase in cell mass, the four clones also failed to grow (Fig. 6B). Ten days after nitrogen deprivation, cultures from two clones bearing the inserted cassette in each of the two possible orientations were visualized by light microscopy, showing the presence of abundant cell debris and few filaments as compared to the WT (Fig. 6C). Nonetheless, heterocysts were observed in the two mutant cultures as in the WT culture, and the frequency of heterocysts was similar in the three cultures, albeit slightly higher in the mutants than in the WT at 24 h and slightly lower at 48 h (Fig. S4). Furthermore, filaments of Δ*kaiABC* strains exhibited a significantly lower autofluorescence intensity under prolonged nitrogen deprivation relative to the wild-type strain (568 ± 156 versus 1,320 ± 84 a.u., mean ± SE, respectively, measured 5 days following nitrogen deprivation), precluding the accurate detection of the decay of autofluorescence in incipient heterocysts. These observations collectively show that deletion of the *kai* genes leads to failure in diazotrophic growth while allowing heterocyst differentiation.

### Candidate genes linking the circadian clock to the behavior of *Anabaena* under nitrogen deprivation

To shed light on the relationship between temporal control of differentiation and the failure in diazotrophy of Δ*kaiABC* strains on one hand and the circadian clock on the other, we searched bioinformatically the *Anabaena* genome for a conserved signature of the RpaA-binding motif previously reported for 101 sequences in *S. elongatus* (4), taking advantage of the 96% similarity between the amino acid sequences of the *S. elongatus* and *Anabaena* RpaA proteins (*Anabaena* RpaA is ORF All0129). Our search was restricted to detect the motif only within regions starting upstream to putative TSSs of genes, based on reported annotations of TSS (25) (motifs within a window of −500 bp to +50 bp relative to the TSS).

A number of genes encoding regulatory proteins were detected among 81 genes bearing putative RpaA binding sites with a FIMO q-value <0.05 (Table S1). These include the ferric uptake regulator-related protein FurC (Alr0957), which is a protein with multiple effects, which affects nitrogen fixation in *Anabaena* (33); the cAMP-binding transcriptional regulator Alr2325 (34); and the RNA polymerase sigma factor SigE, which is involved in expression of late heterocyst-specific genes (35). Additionally, four other transcriptional regulators including a transcriptional regulator of unknown function (Alr3646) and three two-component regulators (All3822, Alr5272, and Alr5069) were detected. It will be of interest to investigate in the future whether these regulators are indeed involved in circadian clock-related activities.

## DISCUSSION

Cells in *Anabaena* filaments exhibit robust circadian rhythms under both nitrogen-replete and nitrogen-deficient conditions. However, the single-cell observations reported here demonstrate that the behavior under both conditions differs considerably. Rather than displaying the high synchrony and spatial coherence characteristic of filaments under nitrogen-replete conditions (13), filaments under nitrogen deprivation display noticeable differences in the phase of expression of a clock-controlled gene between different vegetative cell intervals, when compared to the phase synchrony within the interval. The physiological changes involved in the differentiation of a cell into a heterocyst entail alteration of cell-cell communication, breaking the symmetry of intercellular transfer: heterocysts become a sink of carbohydrates supplied by their vegetative neighbors, whereas heterocysts supply fixed nitrogen products to the neighboring vegetative cells (9). The discoordination between vegetative filaments together with the reduced communication between vegetative cell intervals suggests that a vegetative cell interval and its delimiting heterocysts are the organismic unit in *Anabaena* under nitrogen fixing conditions, whereas the full filament is the organismic unit under nitrogen-replete conditions. This is supported by the loss of spatial coherence of the expression of a clock-controlled gene between vegetative cell intervals along filaments as well as the lower gene expression noise level within intervals as compared to the noise of all vegetative cells along a filament. Discoordination in differentiated filaments also manifested itself in reduced synchrony as measured by the synchronization index between individual vegetative cells located at different vegetative intervals, as compared to cells separated by a similar distance in undifferentiated filaments (13) (Table 1).

A number of cellular processes have been reported to be regulated by circadian clocks in cyanobacteria. For example, the cell cycle is gated both in unicellular *S. elongatus* (6, 7) as well as in *Anabaena* (13). Similarly, experimental evidence supports the notion that the competence state in *S. elongatus* is regulated by the circadian clock (8). Here, we found that differentiation of vegetative cells into heterocysts takes place preferentially in a narrow window of the circadian cycle, suggesting that the circadian clock also gates heterocyst differentiation, and that Δ*kaiABC* strains are impaired in diazotrophic growth. The fitness benefit of possible gating differentiation by the circadian clock could result from minimizing the metabolic load on the cell by avoiding differentiation during periods in which the cell is engaged in other processes that may compete with it. This notion is consistent with our observation that most differentiation events occur primarily when cell division events are infrequent (13). The possibility that cell division of mother cells is a requirement for heterocyst differentiation after nitrogen step-down was previously studied (36). However, a mechanism for the coordination between cell division and differentiation in *Anabaena* has recently been reported (37). The fact that the phase of the clock in a heterocyst is coincident with the phase of the clock of the progenitor’s sister cell indicates that while the clock may gate differentiation, the clock itself is rather insensitive to the differentiation process, despite the attendant metabolic changes involved in the differentiation process.

Interestingly, the absence of a clock does not prevent differentiation of (non-functional) heterocysts. A clue that may point to a mechanism behind the possible gating of differentiation by the circadian clock is furnished by the 5- to 10-fold reduction in the levels of *pecB* transcription in heterocysts relative to vegetative cells. Oscillations are transmitted from the core clock to the *pecBACEF* operon most probably by the master transcription factor RpaA (13). Here, we found that upstream of *pecB*, there are two putative RpaA binding sites (*P* value = 1.5 × 10^−5^, Table S1). Since neither the abundance of RpaA nor its transcription decreases as a result of nitrogen deprivation (18, 38), lower levels of *pecBACEF* transcription may be effected by a reduction in the levels of the active phosphorylated form (RpaA~P), which may be mediated by SasA and CikA in *Anabaena* as in *S. elongatus* (39). SasA is regulated in *S. elongatus* by the phycobilisome-associated B protein (RpaB), which is involved in the integration of temporal and environmental information and stress (40). The conservation of the corresponding genes lends support to these notions (10). Together, these considerations suggest that a link between the circadian clock and the heterocyst differentiation network may be gleaned from the set of genes whose expression is regulated by RpaA~P.

Our observation of circadian oscillations in the transcriptional activity of *pecB* in individual heterocysts, together with the possible inheritance of the phase of oscillation from the primordial vegetative cell, leads us to posit that the circadian clock continues to function in the heterocyst and that rhythmic transcription in the heterocyst is not induced indirectly by time-dependent intercellular signals from clocks in neighboring vegetative cells. While photosystem II is altered in heterocysts (41), photosystem I continues to function (41), and the oscillatory behavior of transcriptional activity of *pecB,* even if smaller, suggests that the circadian clock may modulate photosynthetic activity. The sequential turnoff of gene expression according to position along a vegetative cell interval is characterized by timescales that are considerably longer that those typical of intercellular transport of metabolites (42), which help maintain filaments in homeostasis.

The possible gating of differentiation by the circadian clock and the failure of diazotrophy of Δ*kaiABC* strains led us to investigate the relationship between these two processes using bioinformatics methodologies. The high conservation of circadian clock components among cyanobacteria (10) and, in particular, the high similarity between the protein sequences of RpaA in *Anabaena* and in *S. elongatus* suggested that RpaA function is conserved as a master clock output regulator. Therefore, we looked for the presence of RpaA putative binding sites upstream of *Anabaena* genes, with low FIMO q-values (i.e., high significance). We found that some of the ChIP-validated genes in *S. elongatus* (4) have orthologs in *Anabaena* and have putative binding sites of RpaA. Together, these findings support the notion that RpaA may play a functional role as master regulator of clock outputs in *Anabaena* as in *S. elongatus*. However, we have also identified in *Anabaena* putative RpaA controlled genes with specific roles in heterocyst function, which would explain the lack of heterocyst activity in Δ*kaiABC* strains. In summary, our work revealed that, in *Anabaena*, the circadian clock is further necessary to confront nitrogen stress.

## Data Availability

Source data files and Matlab code have been deposited in Dryad.

## SUPPLEMENTAL MATERIAL

Supporting information msystems.00700-23-s0001.pdf Fig. S1-S4, Table S1 and S2 captions, and Movie 1 legend.

Table S1 msystems.00700-23-s0002.xlsx Scatterplots of the fluorescence intensity from PpecB-gfp expression in adjacent cell pairs along filaments of wild-type background under nitrogen-replete conditions.

Table S2 msystems.00700-23-s0003.xlsx BLASTP analysis of *S. elongatus* orthologous proteins to *Anabaena* sp. strain PCC 7120.

Movie S1 msystems.00700-23-s0004.mp4 Circadian oscillations in *Anabaena* filaments under nitrogen-poor conditions as a function of time.

ASM does not own the copyrights to Supplemental Material that may be linked to, or accessed through, an article. The authors have granted ASM a non-exclusive, world-wide license to publish the Supplemental Material files. Please contact the corresponding author directly for reuse.
