# Supplementary material for: Spatio-temporal coherence of circadian clocks and temporal control of differentiation in Anabaena filaments
Source: mSystems. 2023 Dec 11;9(1):e00700-23. doi: 10.1128/msystems.00700-23 (PMC10805033; doi:10.1128/msystems.00700-23)
Supplement: Supporting information — Fig. S1-S4, Table S1 and S2 captions, and Movie 1 legend. [file msystems.00700-23-s0001.pdf]

Supporting Information

Spatio-temporal coherence of circadian clocks and temporal control of differentiation in *Anabaena* filaments

Rinat Arbel-Goren, Bareket Dassa, Anna Zhitnitsky, Ana Valladares, Antonia Herrero, Enrique Flores and Joel Stavans

Figure S1. Scatterplots of the fluorescence intensity from P_{pecB}-*gfp* expression in adjacent cell pairs along filaments of wild-type background under nitrogen-replete conditions.

Figure S2. Noise of fluorescence intensity of the expression of a clock-controlled gene in *Anabaena*.

Figure S3. Genetic structure in the ∆*kaiABC* mutants.

Figure S4. Heterocyst formation in the ∆*kai* mutants of *Anabaena* sp. strain PCC 7210.

Movie 1. Circadian oscillations in *Anabaena* filaments under nitrogen-poor conditions as a function of time.

Table S1. RpaA putative binding motifs identified in *Anabaena* sp. strain PCC 7120 including FIMO statistics and additional annotations.

Table S2. BLASTP analysis of *S. elongatus* orthologous proteins to *Anabaena* sp. strain PCC 7120.

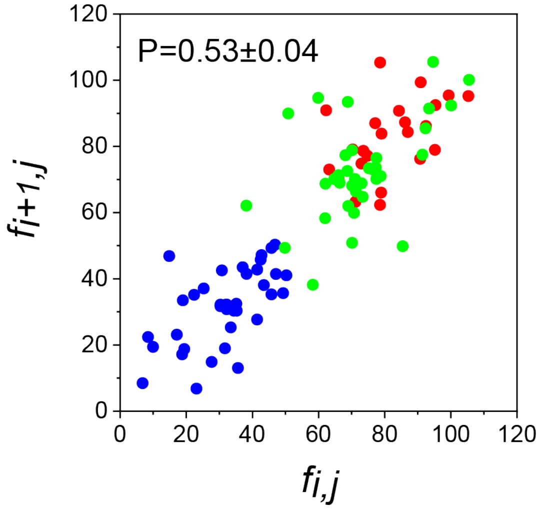

Figure S1. Scatterplot of the fluorescence intensity from P_{pecB}-*gfp* expression in adjacent cell pairs along filaments under nitrogen-replete conditions. The Pearson coefficient P represents an average over the three independent contiguous vegetative stretches of 10 cells and the error represents a standard error. The three different colors code for data from the independent vegetative stretches. Data from Arbel-Goren et al (13).

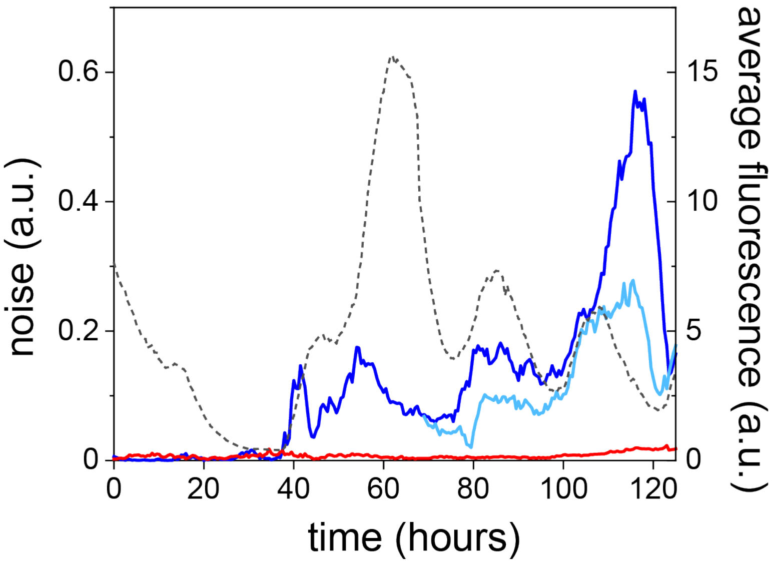

Figure S2. Noise of fluorescence intensity of the expression of a clock-controlled gene in *Anabaena*. Temporal dependence of the cell-cell variability CV^2 (noise, left axis) of the expression intensity of P_{pecB}-*gfp* for vegetative cells along a filament (blue) or within individual vegetative intervals (light blue); Noise of autofluorescence as a function of time (red); Fluorescence intensity from $P_{pecB\text{-}gfp}$ in a filament as a function of time for a wild-type genetic background under nitrogen-poor conditions (dashed black line, right axis).

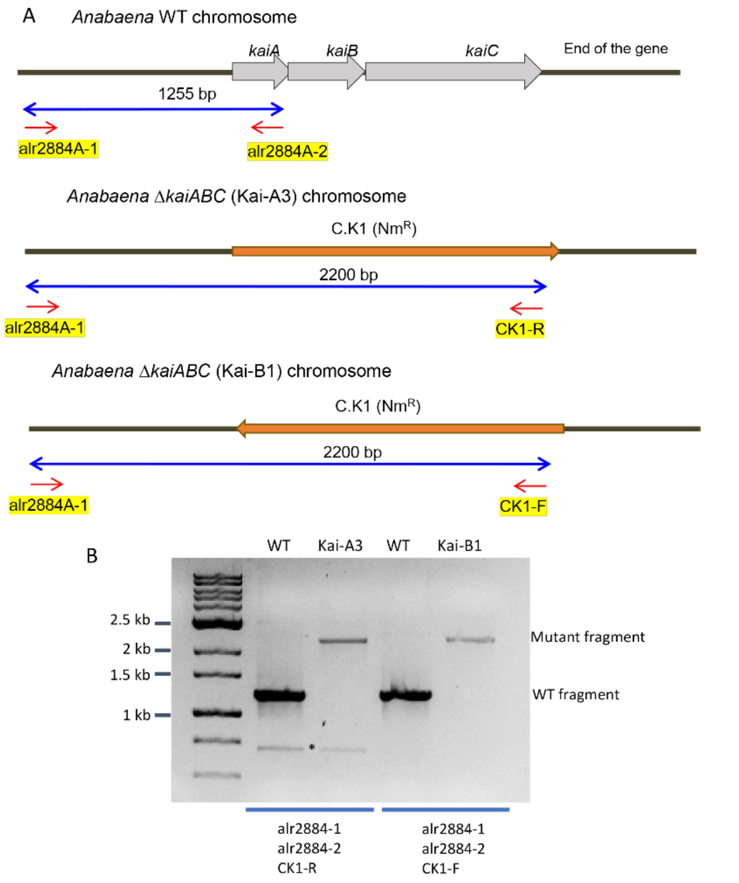

Figure S3. Genetic structure in the Δ*kaiABC* mutants. The Δ*kaiABC* mutants were re-isolated following the procedure described in Arbel-Goren *et al*., 2021, but now the gene-cassette, C.K1, was inserted in both orientations (direct orientation, mutants A; opposite orientation, mutants B). (A) The genetic structure in the *kai* genomic region is shown for the wild type (top scheme), a mutant with the gene cassette in direct orientation (middle) and a mutant with the gene cassette in opposite orientation (bottom). Oligonucleotide primers used in PCR analysis are indicated. (B) PCR analysis with genomic DNA isolated from the wild type or the A3 and B1 *kai* mutants grown in BG11 medium (with neomycin at 20 µg mL^{-1} for the mutants) and incubated for 48 h in $BG11_0$ medium (without neomycin). Three primers were added to each reaction, as indicated, resulting in amplification of only WT DNA fragments in the wild type and only mutant fragments in the mutants, indicating segregation of the mutant chromosomes. *, non-specific amplification product. Oligodeoxynucleotide primer sequences (5’→3’): Alr2884A-1, GCC AGA GTA CTT GTT TCT AAG CAA C; Alr2884A-2, CAG GGT GAG GCG ATA ATC CAT; CK1-F, GCT GCT AAA GGA AGC GGA A; CK1-R, CGA TTC CGA AGC CCA ACC T.

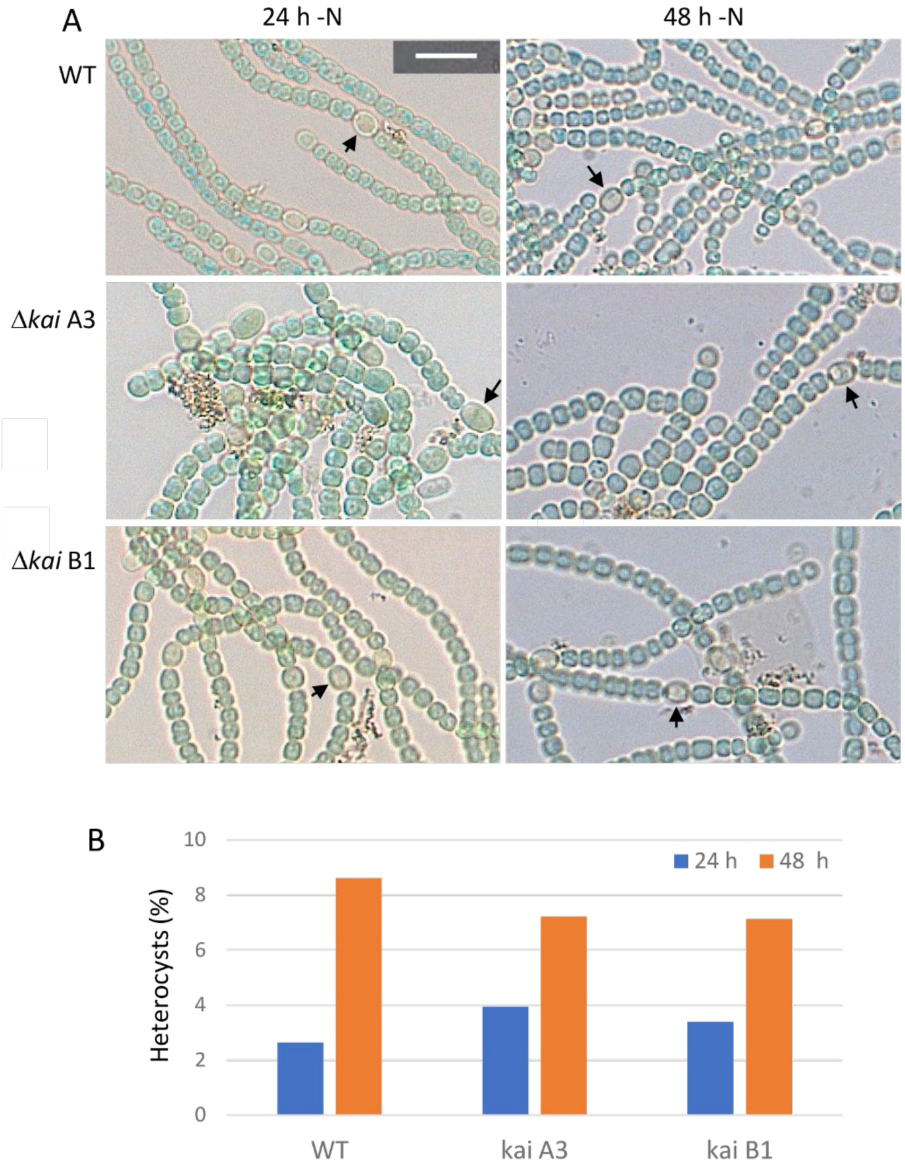

Figure S4. Heterocyst formation in the Δ*kai* mutants of *Anabaena* sp. strain PCC 7210. The strains were grown in BG11 medium (in the presence of neomycin at 20 µg mL^{-1} for the mutants), washed with $BG11_0$ medium and incubated in liquid $BG11_0$ medium (without antibiotic) under photoautotrophic culture conditions (continuous light) for 24 and 48 h, respectively. (A) Examples of filaments showing the presence of heterocysts (some indicated by black arrows). Size bar, 10 µm; same magnification in all the micrographs. (B) Heterocysts as percentage of total number of cells in the three strains after 24 or 48 h of incubation in $BG11_0$ medium. Total number of cells counted: 1300 to 1500 in the 24-h samples; 1000 to 1100 in the 48-h samples.

Movie 1. Circadian oscillations in *Anabaena* filaments under nitrogen-poor conditions as a function of time. (*Left*) Phase contrast images of a filament of an *Anabaena* strain bearing a P_{pecB}-*gfp* promoter fusion, growing under nitrogen-poor conditions. (*Middle*) Autofluorescence as a function of time of the same *Anabaena* filament. (*Right*) GFP fluorescence of the same *Anabaena* filament growing under nitrogen-poor conditions. Images were taken every 30 minutes and time 0 corresponds to the time at which filaments were placed under the microscope. Selected timestamps are indicated at the same times as for Fig. 1 and arrows denote positions of heterocysts.
